# Reference Gene Validation via RT–qPCR for Human iPSC-Derived Neural Stem Cells and Neural Progenitors

**DOI:** 10.1007/s12035-019-1538-x

**Published:** 2019-03-29

**Authors:** Justyna Augustyniak, Jacek Lenart, Gabriela Lipka, Piotr P. Stepien, Leonora Buzanska

**Affiliations:** 1 Department of Stem Cell Bioengineering, Mossakowski Medical Research Centre Polish Academy of Sciences, 5 Pawinskiego Str., 02-106 Warsaw, Poland; 2 Department of Neurochemistry, Mossakowski Medical Research Centre Polish Academy of Sciences, 5 Pawinskiego Str., 02-106 Warsaw, Poland; 3 Institute of Genetics and Biotechnology, Faculty of Biology, University of Warsaw, 5a Pawinskiego Str., 02-106 Warsaw, Poland

**Keywords:** human induced Pluripotent Stem Cell (hiPSC), Neural Stem Cells, Neural Progenitor, quantitative real-time Polymerase Chain Reaction (RT-qPCR), Gene expression reference panel, Relative gene expression

## Abstract

Correct selection of the reference gene(s) is the most important step in gene expression analysis. The aims of this study were to identify and evaluate the panel of possible reference genes in neural stem cells (NSC), early neural progenitors (eNP) and neural progenitors (NP) obtained from human-induced pluripotent stem cells (hiPSC). The stability of expression of genes commonly used as the reference in cells during neural differentiation is variable and does not meet the criteria for reference genes. In the present work, we evaluated the stability of expression of 16 candidate reference genes using the four most popular algorithms: the ΔCt method, BestKeeper, geNorm and NormFinder. All data were analysed using the online tool RefFinder to obtain a comprehensive ranking. Our results indicate that NormFinder is the best tool for reference gene selection in early stages of hiPSC neural differentiation. None of the 16 tested genes is suitable as reference gene for all three stages of development. We recommend using different genes (panel of genes) to normalise RT–qPCR data for each of the neural differentiation stages.

**Electronic supplementary material:**

The online version of this article (10.1007/s12035-019-1538-x) contains supplementary material, which is available to authorized users.

## Introduction

In order to fully understand the processes of neural differentiation, it is necessary to use quantitative real-time polymerase chain reaction (RT–qPCR) for the accurate determination of the relative levels of transcripts of interest. This requires normalisation using a reference gene(s), which show constant expression under the experimental conditions. Suitable reference genes have been identified for many cell types and specific experimental conditions, but the panel of reference genes for human-induced pluripotent stem cells (hiPSC)–derived NSC, eNP and NP populations, has not been described so far. The rationale for selection of putative reference genes in this report was to include the most commonly used and found in the publications referring to different stages of human neural development [1–3].

Human induced Pluripotent Stem Cells (hiPSC) are generated from somatic cells by the introduction of a combination of pluripotency-associated genes such as *POU5F*1, *SOX2* and *NANOG*. hiPSC have the potential to differentiate into any desired cell type, including neurons [4, 5]. The process of reprogramming and differentiation are accompanied by many changes (proteomic, genetic, epigenetic and metabolic) [5–8]. During the neural differentiation process of human hiPSC, different stages of neural development distinguished by specific gene expression profile are generated. In first steps, neuroectoderm-like structures resembling “rosetts” appear, which in suspension culture can form aggregates called neurospheres. At this stage of development neural stem cell (NSC) markers: PAX6, SOX1 and Nestin (NES) are expressed [9]. During further steps of differentiation, iPSC-derived neural precursor cells and neurons thoroughly change their gene expression patterns: some genes are activated, while the expression of others decreases, or are completely switched off. Early neural progenitors (eNP) which are still expressing NES, but also TUBB3 and other early neural markers represent the developmental stage between NSC and neural progenitors (NP). The NP population is characterised by the increased expression of neuronal markers (e.g. TUBB3 and MAP2) and appearance of glial markers [10]. These changes are accompanied by the neurite outgrowth and a proliferation rate decrease [8, 10–13], while in the further differentiation process in the defined media—mature, functional neurons are obtained [9].Thus, eNP and NP are the intermediate stages of neural development between NSC and mature neurons.

Therefore, the aims of this study were to assess the stability of expression for *ACTB*, *CAPN10*, *CCNG1*, *EEF1A1*, *EID2*, *GAPDH*, *HPRT1*, *MYC*, *NAT1*, *PHB*, *RABEP2*, *RPLP0*, *TBP*, *TUBB3*, *UBC* and *ZNF324B* in hiPSC during neural differentiation to NSC, eNP and NP populations. The stability of expression of 16 candidate genes was evaluated by use of 4 statistical algorithms: geNorm [14], NormFinder [15], BestKeeper [16] and the comparative ΔCt method [17]. In our analysis, two approaches have been used; at first, we searched for the most stable reference genes for all developmental stages (NSC, eNP, NP) separately, in the second—for all stages together.

## Materials and Methods

### Cell Culture

The induced pluripotent stem cells (hiPSC) feeder-free cell line was derived from the CD34+ fraction of human cord blood cells by transfection with the EBNA-based episomal system composed of seven factors: SOX2, OCT4, KLF4, MYC, NANOG, LIN28 and SV40L T (The Gibco® Human Episomal iPSC Line, Life Technologies). The process of hiPSC culture and neural differentiation has been described in detail previously [10]. The phenotype of hiPSC-derived NSC, eNP and NP was confirmed qualitatively and quantitatively by immunocytochemistry staining, RT-PCR and RNA-seq [18].

### Gene Ontology Enrichment Analysis

To further explore the functional properties of the analysed genes (*ACTB*, *GAPDH*, *HPRT1*, *TUBB3*, *EID2*, *CAPN10*, *RABEP2*, *ZNF324B*, *NAT1*, *TBP*, *PHB*, *UBC*, *CCNG1*, *MYC*, *EEF1A1*, *RPLP0*): functional protein association networks and Gene Ontology (GO) enrichment analysis were prepared in the STRING: functional protein association networks software (https://string-db.org/). Candidates for reference genes have been classified (FDR < 0.01) in accordance with the following: biological process (GO); molecular function (GO), cellular component (GO); and KEGG pathway. A summary of the description of the tested genes was prepared directly from GeneCards®: The Human Gene Database, UniProtKB/Swiss-Prot Database (https://www.genecards.org/), and STRING: functional protein association networks (https://string-db.org/). Detailed analysis was presented in the Supplementary data_1.

### Design and Specificity of the Primers

All primers were designed using Primer3 software (http://bioinfo.ut.ee/primer3-0.4.0/) based on RNA or DNA sequences found in GenBank (https://www.ncbi.nlm.nih.gov/genbank/). If possible, only primers spanning an exon-exon junction were selected. All of the primers for the candidate genes were chosen according to the general rules of RT-qPCR primer design. In addition, all designed primer pairs were checked for nonspecific amplification by *in-silico* PCR (UCSC, http://genome.ucsc.edu) and by performing a Primer-BLAST search (http://www.ncbi.nlm.nih.gov/tools/primer-blast/). uMELT (*Melting Curve Predictions Software*) was used for melting curve prediction (https://www.dna.utah.edu/umelt/umelt.html). Each pair of primers was confirmed to identify only one specific PCR amplicon with a known expected length. Melting curve analysis showed a single peak for all of the primer pairs, which further confirmed their specificity. All primers were synthesised in the Laboratory of DNA Sequencing and Oligonucleotide Synthesis, Institute of Biochemistry and Biophysics, Polish Academy of Sciences (http://oligo.pl/). The primer details for all 16 candidate reference genes are shown in Table 1 [10–12].

**Table 1:** Candidate reference genes, primer sequences and characteristics of RT-qPCR amplicons in hiPSC at the different stages of neural develoment

Gene symbol	Gene description	Genebank accession Number	Gene function	Primer sequences (5′-3′) (forward/reverse)	Primer Tm (°C)	Amplicon length (bp)	Product efficiency *E* (%)
*ACTB*	*Homo sapiens* actin beta	NM_001101.3	Cytoskeletal structural protein	GCTCACCATGGATGATGATATCGC	61.74	169	1.95
				CACATAGGAATCCTTCTGACCCAT	59.90		
*GAPDH*	*Homo sapiens* glyceraldehyde-3-phosphate dehydrogenase	NM_002046.5	Oxidoreductase in glycolysis and gluconeogenesis	GTTCGACAGTCAGCCGCATC	61.69	90	2.12
				TCCGTTGACTCCGACCTTCA	60.82		
*HPRT1*	*Homo sapiens* hypoxanthine phosphoribosyltransferase 1	NM_000194.2	Purine metabolism	AGGCGAACCTCTCGGCTTTC	62.51	166	1.92
				CTGGTTCATCATCACTAATCACGAC	59.77		
*TUBB3*	*Homo sapiens* tubulin beta 3 class III. transcript variant 1	NM_006086.3	Cytoskeletal protein	CAACCAGATCGGGGCCAAGTT	62.95	146	2.03
				GAGGCACGTACTTGTGAGAAGA	60.03		
*EID2*	*Homo sapiens* EP300 interacting inhibitor of differentiation 2	NM_153232.3	Cell differentiation	GGCATCGCTCTGTCCAGTTA	59.82	74	2.14
				GCTTGGACATCTCAGACCGT	59.75		
*CAPN10*	*Homo sapiens* calpain 10	NM_023083.3	Cytoskeletal remodelling and signal transduction	TCTCACCGGGCTACTACCTG	60.04	86	2.05
				CCCGGTAGAGAAGACTCGGA	60.11		
*RABEP2*	*Homo sapiens* rabaptin. RAB GTPase binding effector protein 2	NM_024816.2	Growth factor activity, GTPase activator activity. Endocytosis. Protein transport	AGGAAGGGGCAAATGGTGAG	59.96	96	2.08
				CAGCCTTCATGGTTTCCATTTCTG	60.62		
*ZNF324B*	*Homo sapiens* zinc finger protein 324B	NM_207395.2	Regulation of transcription	CATTGGAAGGACAAACCTAGGATGATG	61.81	164	1.93
				CTTATCTGCTCCAAAGCTATCACTGTC	61.63		
*NAT1*	*Homo sapiens* N-acetyltransferase 1	NM_001160170.3	4Metabolic pathways	TGGTTGCCGGCTGAAATAAC	59.11	93	2.09
				TCTGTCTAGGCCAGTCTCCT	59.00		
*TBP*	*Homo sapiens* TATA-box binding protein	NM_003194.4	General RNA polymerase II transcription factor	GCAAGGGTTTCTGGTTTGCC	60.25	80	2.14
				CAAGCCCTGAGCGTAAGGTG	61.02		
*PHB*	*Homo sapiens* prohibitin	NM_001281496.1	Transcriptional modulation	TGGAAGCAGGTGAGAATGGAG	59.72	76	2.05
				ATCATGGAGCAGAGGAGGACT	60.06		
*UBC*	*Homo sapiens* ubiquitin C	NM_021009.6	Protein degradation	ACGGGACTTGGGTGACTCTA	59.89	82	2.15
				ATCGCCGAGAAGGGACTACT	60.11		
*CCNG1*	*Homo sapiens* cyclin G1	NM_004060.3	Cell cycle regulation, growth regulation	GCCTCTCGGATCTGATATCGT	58.91	138	2.04
				CATTCAGCTGGTGTAGCAGT	57.89		
*MYC*	*Homo sapiens* v-myc avian myelocytomatosis viral oncogene homolog	NM_002467.4	Transcription factor, oncogene	CCCTCCACTCGGAAGGACTA	60.03	96	2.07
				GCTGGTGCATTTTCGGTTGT	59.97		
*EEF1A1*	*Homo sapiens* eukaryotic translation elongation factor 1 alpha 1	NM_001402.5	Translation elongation factor	TGTTCCTTTGGTCAACACCGA	60.07	122	2.07
				ACAACCCTATTCTCCACCCA	57.95		
*RPLP0*	*Homo sapiens* ribosomal protein lateral stalk subunit P0	NM_001002.3	Ribosomal proteins	CCTCGTGGAAGTGACATCGT	59.76	76	2.10
				CTGTCTTCCCTGGGCATCAC	60.39		

### RNA Extraction and cDNA Synthesis

Total RNA extraction including DNase treatment (Clean-Up RNA Concentrator kit, A&A BIOTECHNOLOGY, Gdynia, Poland) was carried out using the Total RNA Mini Kit (A&A BIOTECHNOLOGY, Gdynia, Poland) according to the manufacturer’s instructions. Extracted RNAs were quantified by NanoDrop ND-1000 spectrophotometer (Thermo Fisher Scientific, Waltham, USA) and the absorbance ratios at 260/280 nm and 260/230 nm were measured to control RNA purity. The RNA integrity was checked by electrophoresis in 1.5% agarose gels. Total RNA (2 μg) was reverse transcribed using the High-Capacity RNA-to-cDNA™ Kit (Thermo Fisher Scientific) in a volume of 20 μL, according to the manufacturer’s instructions.

### Quantitative Polymerase Chain Reaction

The RT–qPCR reactions were performed in the LightCycler® 96 (Roche Diagnostics GmbH, Mannheim, Germany). Each reaction was performed in triplicate in 96-well plates (FrameStar® 480/96 for Roche LightCyler, 4titude® Ltd., Wotton, UK) in a reaction volume of 20 μL. All reactions contained 5 μL of cDNA (10 ng), 10 μL of 3color 2× HS-qPCR Master Mix SYBR (A&A BIOTECHNOLOGY, Gdynia, Poland), 1 μL of 10 mM of each primer and 3 μL of DEPC-treated water. The reaction protocol starts with a 3-min initial denaturation step at 95 °C, 40 cycles of 95 °C for 15 s and 60 °C for 60 s. Subsequently, the melting curve was verified by amplification of a single product, which was generated starting at 65 °C and increasing to 99 °C by 1 °C for each 30-s cycle. Each experiment included a no template control. All experiments described in this article were carried out following strict MIQE guidelines [19] with different types of negative controls. To monitor genomic DNA contamination, no reverse transcriptase control (–RT) was used. To avoid sample contamination and primer-dimer formation that could produce false positive results, no template control (-NTC) was used.

### Data Analysis

The quantification cycle (Cq) values were automatically calculated by the qPCR instrument software (LightCycler® 96 Software, Roche Diagnostics GmbH, Mannheim, Germany). The data were analysed using GeneEx 6.1 software (MultiD Analyses AB, Göteborg, Sweden). Different statistical algorithms such as geNorm [14], NormFinder [15], BestKeeper [16] and ΔCt [17] were used to evaluate expression stability from the Cq values of each candidate for reference gene. The RefFinder online tool (http://fulxie.0fees.us/?i=1) was chosen to calculate comprehensive ranking based on the data from abovementioned algorithms [19]. In BestKeeper and ΔCt method analysis, stability of candidate reference genes was calculated directly from raw Cq value, while Cq values were imported to the geNorm and NormFinder software raw after conversion into relative quantities, according to the formula *Q* = *E*^−ΔCq^, in which *E* = amplification efficiency and ΔCq = the corresponding Cq value—minimum Cq. The genes with the lowest standard deviation (SD) and coefficient of variance (CV) were treated as the most stable reference genes. Measures of expression stability (*M* value) for candidate reference genes in geNorm were based on the ratio: the level of pairwise variation for each reference gene with all other control genes and the standard deviation (SD) of the logarithmically transformed expression [14].

## Results

### Selection of Putative Reference Genes

A total of 16 candidate reference genes were selected from the relevant literature related to human neural development [1, 2]. The selection included some of the most frequently used reference genes: *GAPDH*, *TUBB3* and *ACTB*. However, a previous study has shown that these commonly used housekeeping genes (HKGs) are inappropriate in hiPSC due to the substantial variability of their expression during differentiation [3]. The characteristics of candidate reference genes including their function are presented in Table 1 and Supplementary data_1.

### Gene Ontology Enrichment Analysis

To further explore stability of candidates for reference genes (*ACTB*, *GAPDH*, *HPRT1*, *TUBB3*, *EID2*, *CAPN10*, *RABEP2*, *ZNF324B*, *NAT1*, *TBP*, *PHB*, *UBC*, *CCNG1*, *MYC*, *EEF1A1*, *RPLP0*), their functional properties were specified using functional protein association networks and Gene Ontology (GO) enrichment analysis (https://string-db.org/). In the Gene Ontology enrichment analysis (*FDR < 0.01*), candidates for reference genes were assigned to a set of predefined bins, depending on their functional characteristics: biological processes, molecular function and cellular compound. In terms of the biological process at *FDR < 0.01* significance level, the largest groups of candidates for reference genes were classified to the following: primary metabolic process (*ACTB*, *CAPN10*, *EEF1A1*, *EID2*, *GAPDH*, *HPRT1*, *MYC*, *RPLP0*, *TBP*, *TUBB3*, *UBC*, *ZNF324B*); nucleobase-containing compound metabolic process, organic substance biosynthetic process (*EEF1A1*, *EID2*, *GAPDH*, *HPRT1*, *MYC*, *PHB*, *RPLP0*, *TBP*, *UBC*, *ZNF324B*); nucleobase-containing compound biosynthetic process (*EEF1A1*, *EID2*, *HPRT1*, *MYC*, *PHB*, *RPLP0*, *TBP*, *UBC*, *ZNF324B*); RNA biosynthetic process (*EEF1A1*, *EID2*, *MYC*, *PHB*, *RPLP0*, *TBP*, *UBC*, *ZNF324B*); response to chemical (*ACTB*, *CAPN10*, *EEF1A1*, *EID2*, *GAPDH*, *HPRT1*, *MYC*, *NAT1*, *PHB*, *TUBB3*, *UBC*) etc. According to molecular function (GO) gene annotation (*FDR < 0.01*), *RPLP0* was classified as structural constituent of ribosome, while *ACTB*, *RPLP0* and *TUBB3* as protein coding genes with structural molecule activity. In predefined cellular component (GO), *FDR < 0.01* tested genes were classified into the following groups: nucleus (*ACTB*, *CCNG1*, *EEF1A1*, *EID2*, *GAPDH*, *MYC*, *PHB*, *RPLP0*, *TBP*, *TUBB3*, *UBC*, *ZNF324B*); intracellular part (*ACTB*, *CAPN10*, *CCNG1*, *EID2*, *GAPDH*, *HPRT1*, *MYC*, *NAT1*, *PHB*, *RABEP2*, *RPLP0*, *TBP*, *TUBB3*, *UBC*, *ZNF324B*); membrane-bounded organelle (*ACTB*, *CAPN10*, *CCNG1*, *EID2*, *GAPDH*, *HPRT1*, *MYC*, *PHB*, *RABEP2*, *RPLP0*, *TBP*, *TUBB3*, *UBC*, *ZNF324B*); cytosol (*ACTB*, *CAPN10*, *EEF1A1*, *GAPDH*, *HPRT1*, *MYC*, *NAT1 RPLP0*, *UBC*); extracellular exosome (*ACTB*, *GAPDH*, *HPRT1*, *PHB*, *RPLP0*, *TUBB3*, *UBC*)*.* Detailed analysis is presented in Supplementary data_1.

### Expression Profiles of Candidate Reference Genes

**Fig. 1:**
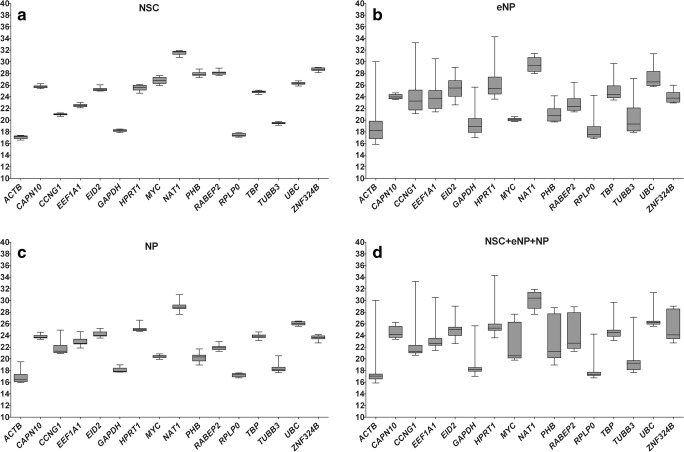
Reference gene Cq value distribution. Box plots of the Cq values in each developmental stage. **a** NSC. **b** eNP. **c** NP. **d** NSC + eNP + NP for all 16 reference genes. The box indicates the 25th and 75th percentiles and the whiskers caps represent the maximum and minimum values. A centre line across the boxes indicates the median. Cq value is the average of 18 measurements

hiPSC cells are derived from one clone and are a very homogeneous population. In the studies described in this manuscript, we used six biological repetitions, and each experiment was performed in triplicate. That means each Cq value is the average of 6 × 3 = 18 repetitions. The absolute Cq values expressed as the mean of average of the replicates (3 independent experiments in 3 replicates and each replicate in 3 RT–qPCR runs). The 25th and 75th percentiles for each candidate reference gene for all studied developmental stages (NSC, eNP, NP) were represented in the form of box plots (Fig. 1). The same data set was analysed separately for each developmental stage (Fig. 1a–c) or for all stages combined (Fig. 1d). The expression levels of 16 tested candidate reference genes were determined using the quantification cycle (Cq) values. The results showed that *ACTB* (Cq = 17.066), *RPLP0* (Cq = 17.499) and *GAPDH* (Cq = 18.212) were the highest expressed genes in the NSC stage. The same set of genes was also expressed on the highest level in eNP and NP stages and when combined data for all three stages were taken into account simultaneously. The lowest expressed genes, with the highest Cq values, were *NAT1* and *UBC*. Each candidate reference gene showed expression variation, but this variation is particularly high at the eNP stage of development (Fig. 1b). However, even at this stage, it is possible to find genes that display stable expression. Box plots are useful for comparing distributions between several groups but without making any assumptions about the underlying statistical distribution or any other analysis. To find genes with the most stable expression, it is necessary to use specialised statistical tools. In our study, we used the most popular computational programs: ΔCt method, BestKeeper, geNorm and NormFinder.

### Reference Gene Ranking Based on the ΔCt Method

**Fig. 2:**
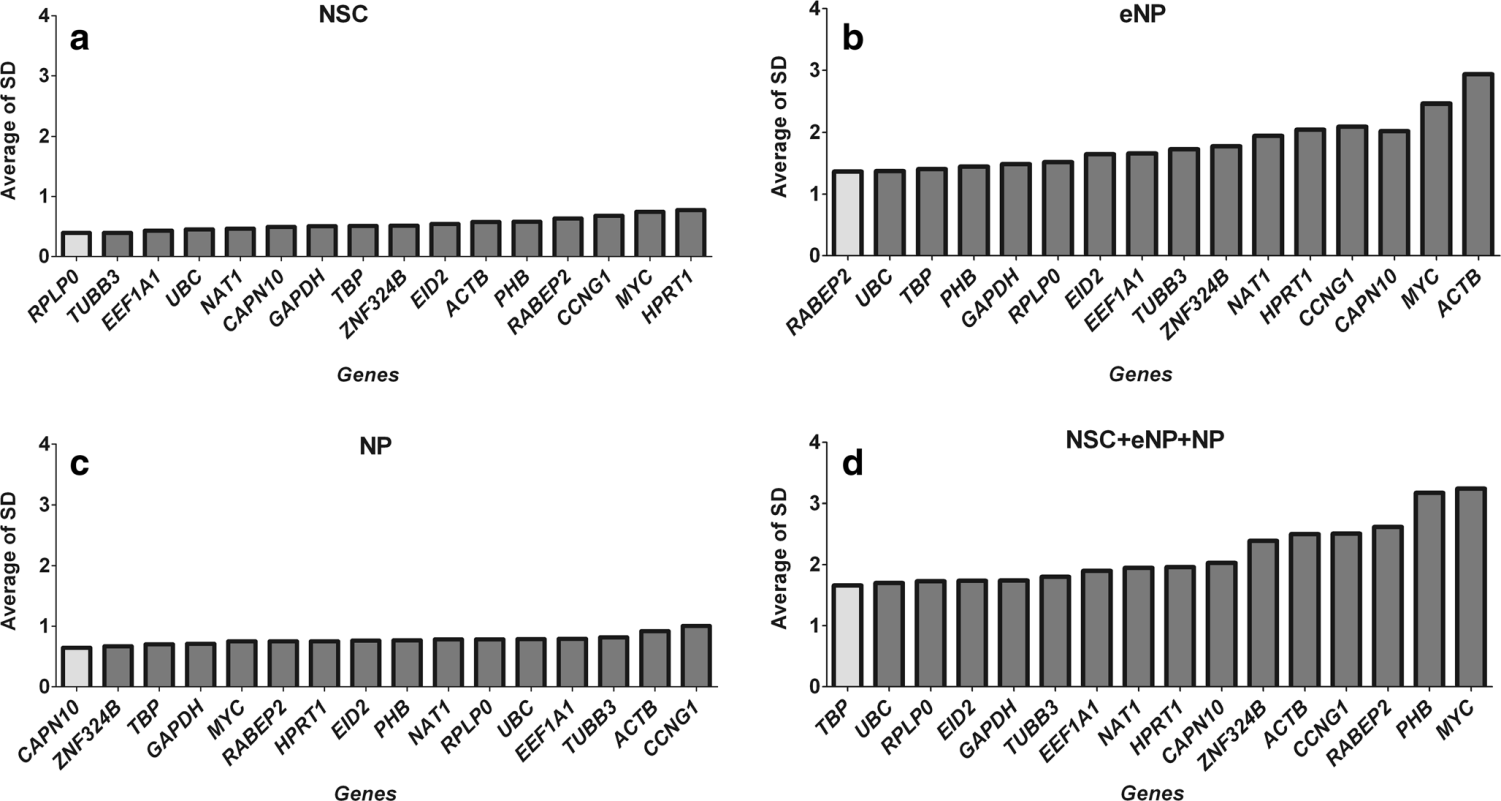
Gene expression stability of 16 potential reference genes calculated by the ΔCt method. **a** NSC. **b** eNP. **c** NP. **d** NSC + eNP + NP. The box in grey indicates expression levels of the most stable genes

The ΔCt method compares relative expression of “pairs of genes” within each sample and takes into account all possible gene combinations [17]. This method shows a different pair of reference genes for each developmental stage, and yet another one for all stages analysed together. The genes with the most stable expression (with the lowest average standard deviation) were *RPLP0*, *TUBB3* for NSC; *RABEP2*, *UBC* for eNP; *CAPN10*, *ZNF324B* for NP and *TBP*, *UBC* for all stages together (Fig. 2). In general, the expression of selected genes appears to be much more stable at NSC and NP stages of development compared to the eNP stage. Due to high variability at the eNP stage, high variability is also observed when analysing data from all three stages together. Even the genes with the most unstable expression in the NSC stage have a lower SD value than those with the most stable expression at the eNP stage (0.774 vs 1.365). On the other hand, the genes with the least stable expression were *HPRT1* for NSC, *ACTB* for eNP, *CCNG1* for NP and finally *MYC* for all three stages.

### Reference Gene Ranking Based on BestKeeper

**Fig. 3:**
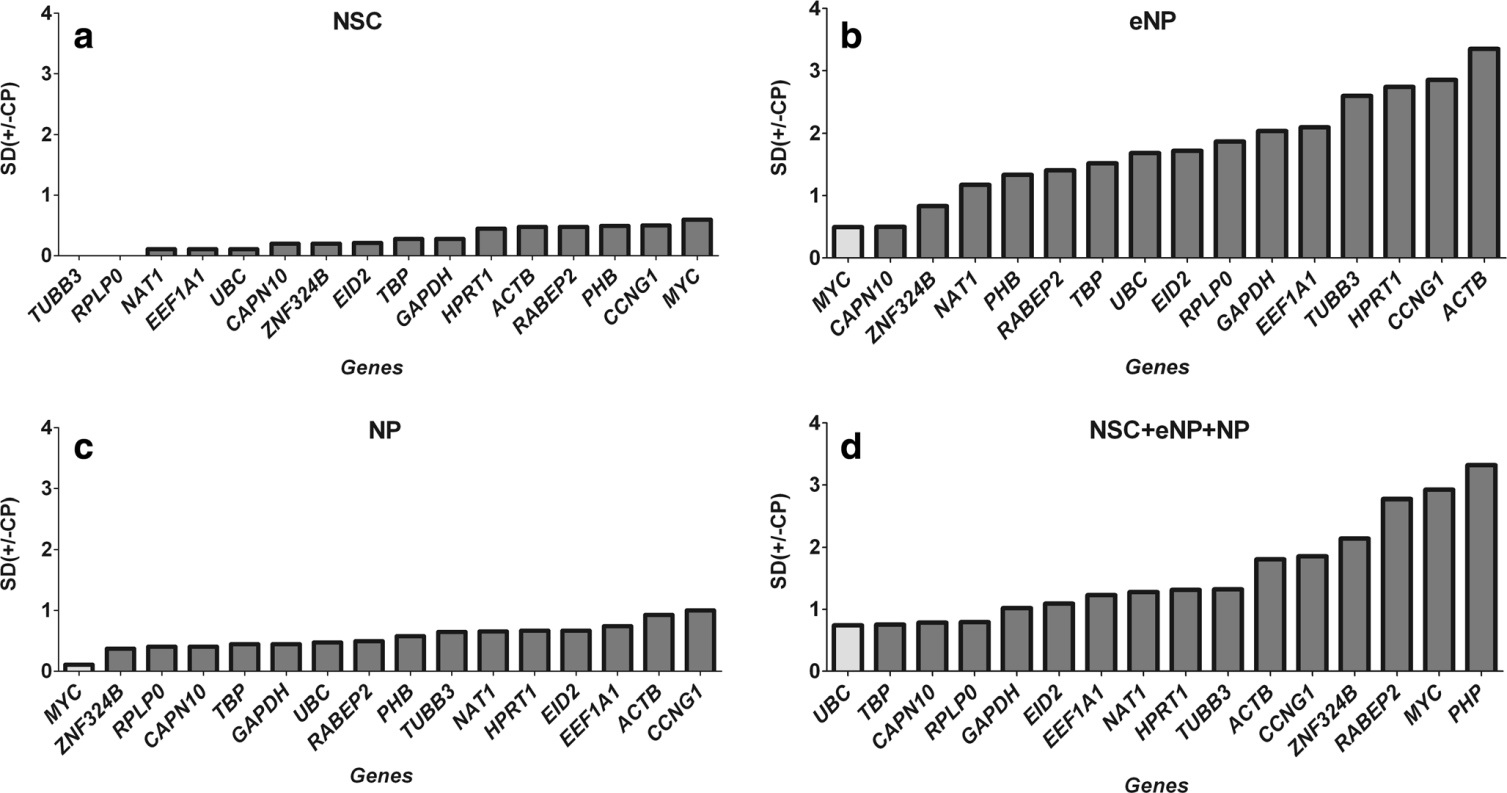
BestKeeper expression stability values. **a** NSC. **b** eNP. **c** NP. **d** NSC + eNP + NP. The box in grey indicates the most stable gene expression level

BestKeeper estimates gene expression stability using two parameters: standard deviation (SD) and coefficient of variation (CP) of each gene in all samples [16]. As in the case of the ΔCt method, BestKeeper found different genes with the most stable expression for each stage of development (Fig. 3). *TUBB3* and *RPLP0* genes were found as the most stably expressed for the NSC stage (SD ( ± CP)) values for these two genes are exactly 0.000, so they are “ideal” reference genes), *MYC*—for eNP and NP stages and the *UBC* gene for all three stages of development. In the case of this algorithm, the order of genes is very interesting. The most stable expression, and therefore the best candidate for the reference gene for the eNP and NP stages (*MYC*), is the worst (with the most unstable expression level) gene in the NSC stage. It is also penultimate in the ranking of all data taken together. The results obtained from BestKeeper did not completely agree with those obtained from the ΔCt method. The reference genes with the last stable expression *ACTB* for the eNP stage and *CCNG1* for the NP stage were ordered exactly like in the ΔCt method.

### Reference Gene Ranking Based on geNorm

**Fig. 4:**
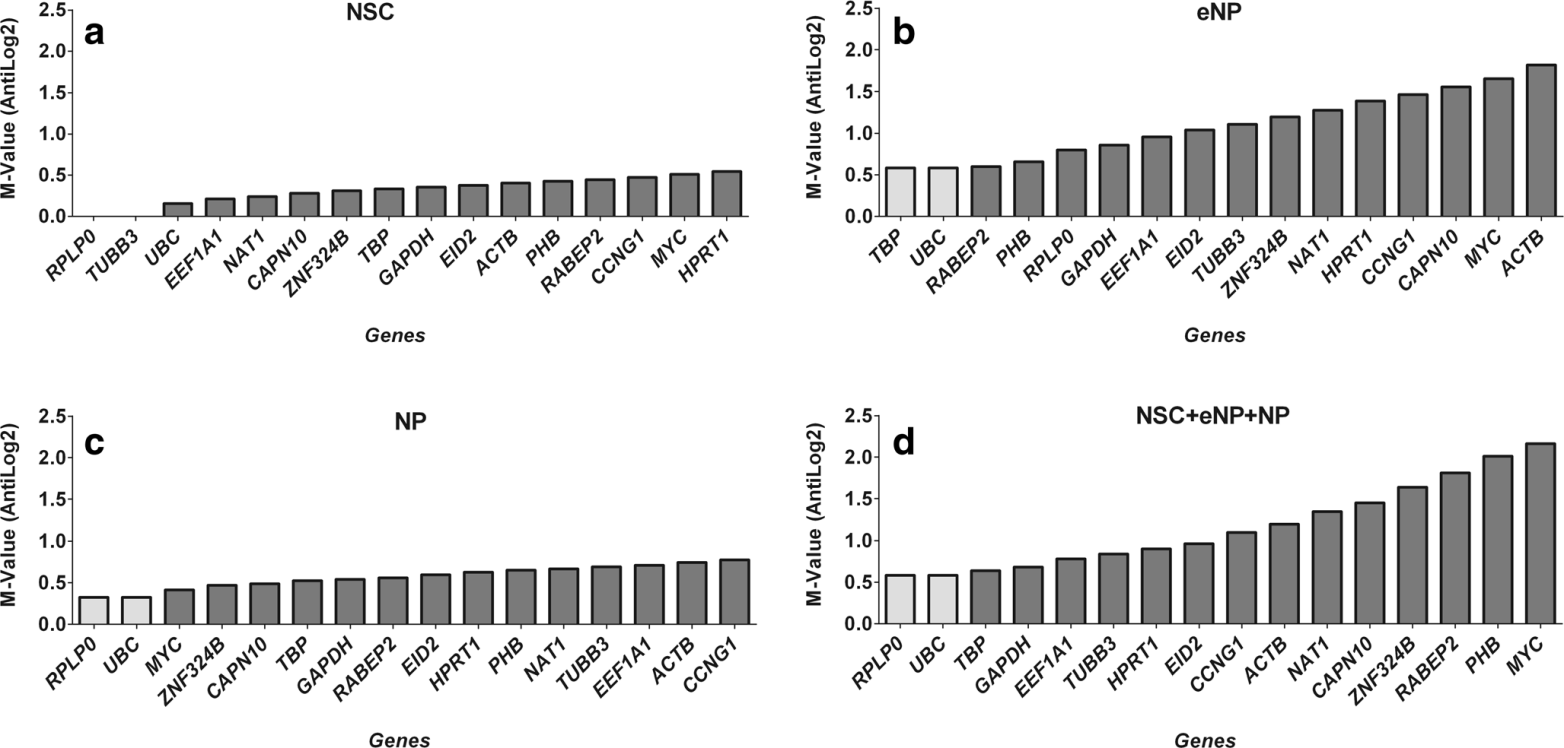
geNorm expression stability values. **a** NSC. **b** eNP. **c** NP. **d** NSC + eNP + NP. Boxes in grey indicate expression levels of the most stable genes

geNorm calculates the stability expression value (*M*) which is the mean pairwise variation for expression of a gene compared with all other tested potential reference genes. The final result of the stepwise exclusion of genes with unstable expression levels is to select two genes with the most stable expression [14]. geNorm analysis of candidate reference genes in the NSC developmental stage showed that *RPLP0* and *TUBB3* had the lowest *M* value of 0.000, suggesting them to be the best and even ideal potential reference genes. In the eNP stage, *UBC* and TBP had the *M* value of 0.583 and displayed stable expression. In the NP stage, *RPLP0* and *UBC* presented stable expression (*M* = 0.323), and finally for all three stages together the best reference genes were *UBC* and *RPLP0* with the stability value of 0.584 (Fig. 4). When using geNorm, it should be kept in mind that it does not correct for co-regulation and the genes from the same metabolic pathway should be avoided. Additionally, one gene *RPLP0* is the most stably expressed for NSC, NP and even for all stages together.

### Reference Gene Ranking Based on NormFinder

**Fig. 5:**
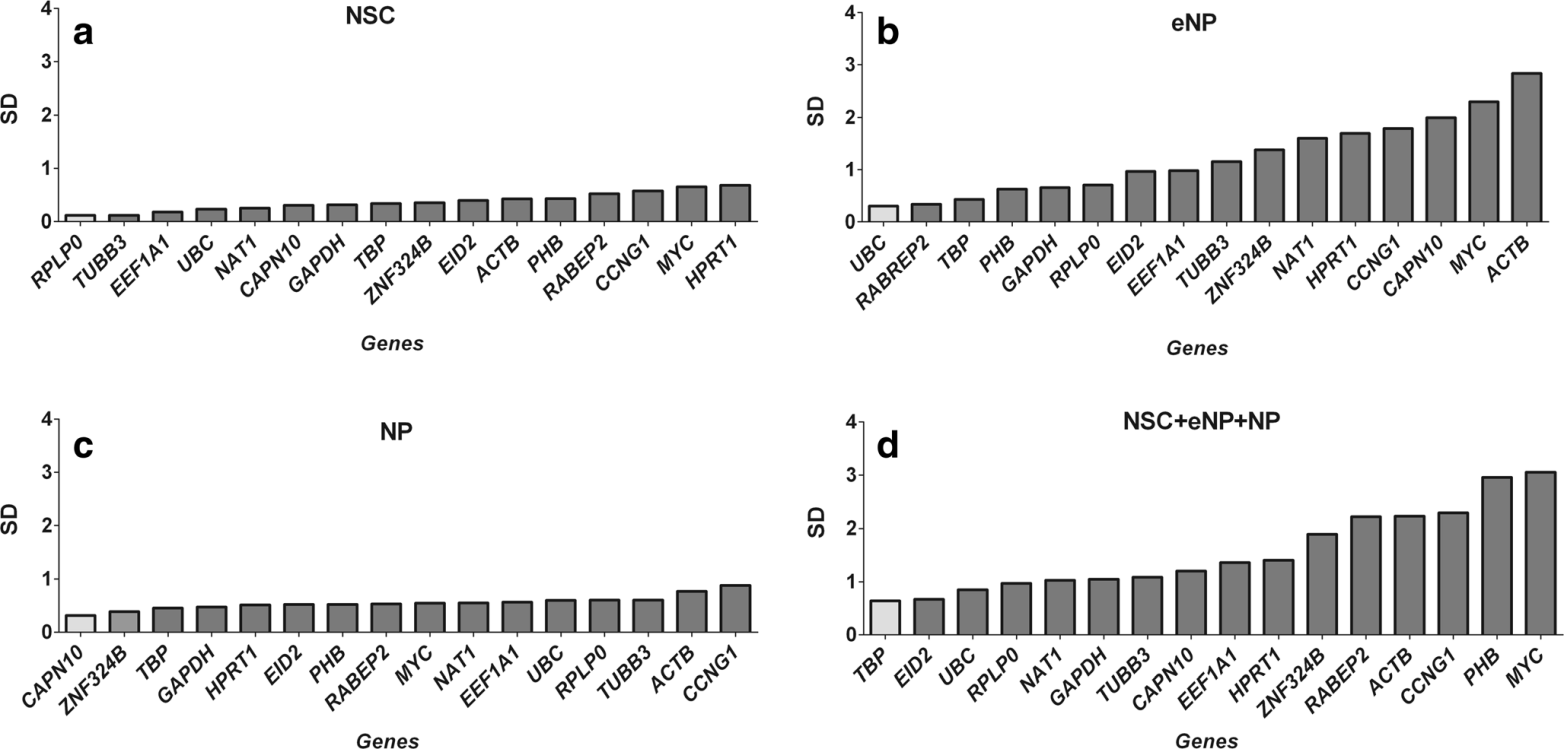
NormFinder expression stability values. **a** NSC. **b** eNP. **c** NP. **d** NSC + eNP + NP. The box in grey indicates expression levels of the most stable genes

NormFinder, in contrast to other algorithms, takes into account the intra- and the inter-group expression variation. It ranks the set of candidate reference genes according to their expression stability in a given sample set and given experimental design. A lower stability value indicates higher expression stability. NormFinder analysis recommended *RPLP0* (*M* value of 0.116) as the most reliable reference gene for the NSC developmental stage, *UBC* (*M* value of 0.302) for the eNP developmental stage, *CAPN10* (*M* value of 0.312) for the NP developmental stage and *TBP* (*M* value of 0.639) for all three stages together (Fig. 5). In the NormFinder analysis, *ACTB*, *MYC* and *CCNG1* were the worst candidates for reference genes. This result was consistent with the result from the ΔCt method, BestKeeper and geNorm.

### Comprehensive Ranking

**Fig. 6:**
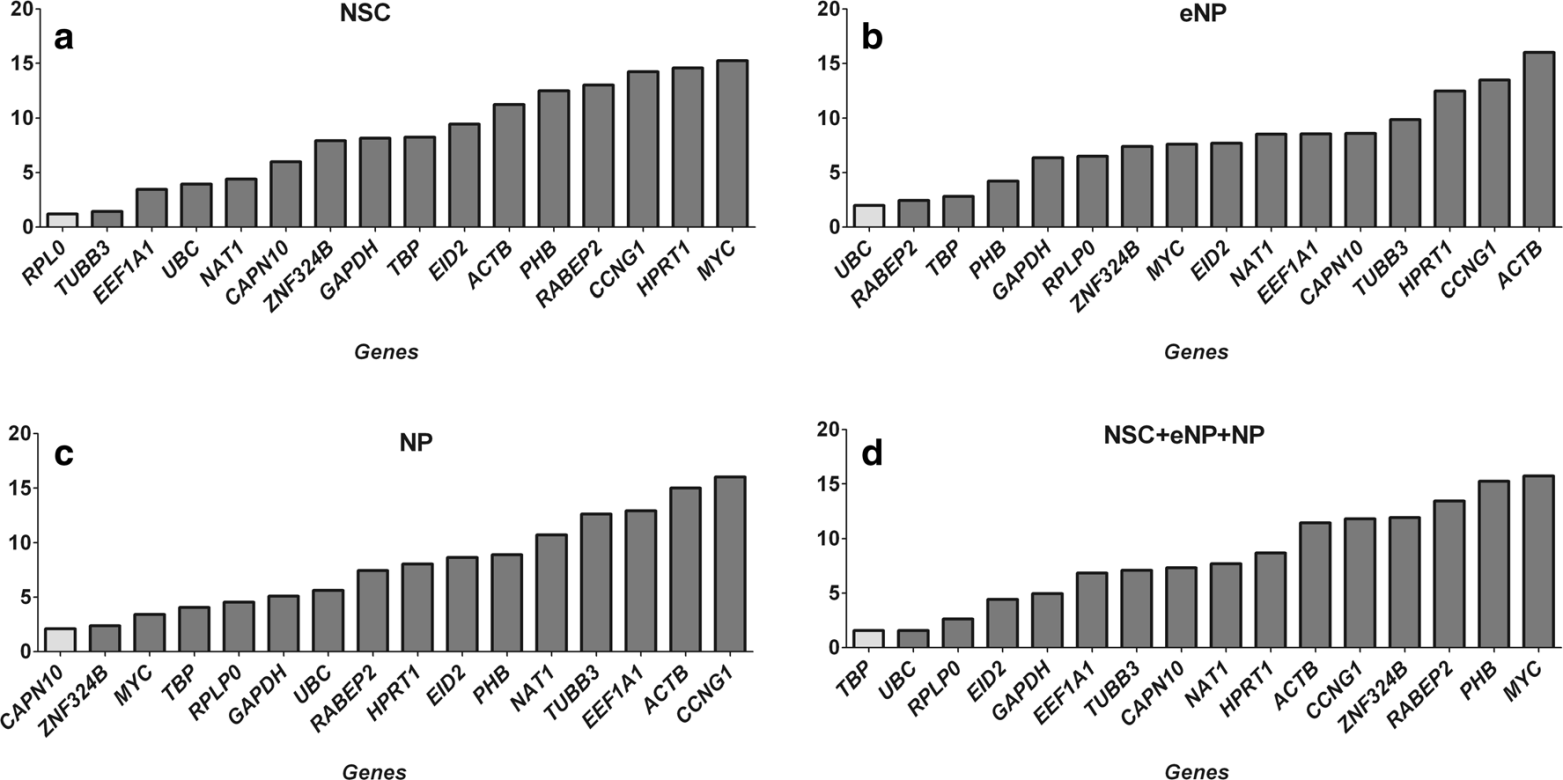
Comprehensive expression gene stability. **a** NSC. **b** eNP. **c** NP. **d** NSC + eNP + NP. The box in grey indicates expression levels of the most stable genes

Taking into consideration advantages and disadvantages of the four different statistical methods described above, we used the RefFinder (a web-based tool) to calculate an overall comprehensive ranking for the best RT–qPCR quantification comparator from all tested 16 potentially reference genes. The algorithm assigns an appropriate weight to an individual gene and calculates the geometric means of their weights for the overall ranking numbers produced by the ΔCt method, BestKeeper, geNorm and NormFinder. Genes with the smallest geometric means were considered to have the most stable level [20]. As shown in Fig. 6, the gene with the most stable expression for the NSC stage was *RPL0*, for the eNP—*UBC*, for the NP—*CAPN10* and for all stages together—*TBP*. The genes with the worst expression stability appeared as *MYC*, *ACTB*, *CCNG1* and again *MYC*, respectively.

### Optimal Reference Gene Numbers

To determine the optimal number of genes required for normalisation, NormFinder software calculates the standard deviation (SD) for each gene as well as the accumulated standard deviation (Acc. SD). The accumulated standard deviation is an excellent indicator of the optimal number of reference genes. Acc. SD reached its lowest value 0.00651 with up to 13 reference genes for the NSC developmental stage. In this case, using only four reference genes, it increases Acc. SD to the level 0.0134. The use of only two reference genes (Acc. SD = 0.0947) should be sufficient in the eNP developmental stage. For the NP developmental stage and for all stages together, the optimal number of reference genes is 14 (Acc. SD = 0.1069) and 10 (Acc. SD = 0.3235), respectively (Supplementary data_2). For the NSC and NP populations, many genes show low variability and therefore they are suitable for reference genes (13 and 14 respectively). In the eNP population, 16 genes show high variability and only two show low variability, and they should be used for normalisation.

## Discussion

The aim of this report was to generate the panel of putative reference genes for NSC, eNP and NP according to gene ontology (GO) *in sillico* and validate these genes in vitro. So far, reference gene validation studies for these stages of human neural development have not been described.

The RT–qPCR method is considered to be the gold standard in gene expression analysis. Correct determination of the reference genes which are used as a comparator is the most important step of gene expression analysis since wrong selection of the reference gene(s) leads to incorrect data analysis. The finding of a reference gene(s) for hiPS cells during neural differentiation is a particularly difficult task. At the molecular level, the differentiation of iPSC towards neural stem cells and neural progenitors is orchestrated by the sequential expression of distinct sets of genes in specific stages of development. However, to generate valid data from such studies, the correct endogenous control reference gene(s) for normalisation of data must be found.

The selected putative reference genes included the most commonly used (*ACTB*, *GAPDH* and *HPRT1*) and found in the publications referring to different stages of human neural development [1–3]. Candidates for reference genes were classified with functional protein association networks software (STRING, https://string-db.org/) by taking into account the following: biological process (GO); molecular function (GO) and cellular component (GO) (Supplementary data_1).

The hiPSC-derived NSC, eNP and NP showed wide variations in housekeeping gene Cq values ranging from 17.00 to 31.50. These wide variations confirm that during neural differentiation, the expression of genes essential for cell metabolism, and therefore used as reference genes, changes dramatically. The arbitrary selection of a single reference gene should be avoided, because these reference genes may be differentially regulated and thus increase the probability of producing false data. The four commonly used algorithms (comparative ΔCt method, BestKeeper, geNorm and NormFinder) to check the potential reference gene expression stability have been employed in this study. Each of the applied methods has led to the selection of different gene as having the most stable expression level. This confirms once again that differentiating cells are a very demanding research model. In simple models, different algorithms give very similar and sometimes even identical results.

Table 3 and Supplementary data_4 summarise the gene expression stability values calculated by all methods for all stages of neural development (separately and together). Regardless of the method used, the lowest value attests to grater reference gene expression stability. The numerical values of gene expression stability are considerably lower for NSC and NP developmental stages than for eNP and NSC + eNP + NP. The other general conclusion can be drawn from Table 3: expression of none of the tested genes is the most stable in all differentiation stages. Gene expression in NSC and NP developmental stages is stable; the difference in stability between the most stable and the most unstable gene expression is very small and is observed regardless of the program used (Fig. 2a, c; Fig. 3a, c; Fig. 4a, c; Fig. 5a, c). At present, there is no standard method for the selection of reference genes. Some researchers, with respect to the geNorm algorithm, suggest that genes with *M* values below 1.5 indicate a good measure of gene level stability [21]. Taking into account this criterion, all examined reference genes at the NSC stage and at the NP stage are good reference genes. Twelve genes at the eNP stage have an *M* value lower than 1.5. BestKeeper calculates the SD and CV based on the Cq values of each reference gene. Genes with an SD value of < 1.0 have a stable expression, and the gene with the lowest SD and CV values was identified as having the most stable expression level [22]. All potential reference genes at the NSC stage of development meet this criterion, as do all with the exception of one at the NP stage. However, only three genes meet such criteria (of the lowest SD and CV values) in the case of the eNP stage. There are no criteria for the comparative ΔCt method as well as the NormFinder algorithm because both use SD values to organise gene expression order from the most stable to the least stable.

Comparison of the positions of individual genes in the ranking (Table 2, Supplementary data_3) shows complete compatibility between NormFinder and Comprehensive Ranking. NormFinder [15] combines the advantages of the three other approaches by estimation of both intra- and inter-group expression variations. It directly and robustly evaluates not only gene expression stability but also calculates the optimal number of reference genes required for normalisation, so it should be preferred to the other methods.

**Table 2:** Reference gene ranking order in hiPSC-derived neural stem cells and neural progenitors

	NSC					eNP					NP					NSC + eNP + NP				
Reference gene	ΔCt	BK	gN	NF	CR	Ct	BK	gN	NF	CR	ΔCt	BK	gN	NF	CR	ΔCt	BK	gN	NF	CR
*ACTB*	11	12	11	11	11	16	16	16	16	16	15	15	15	15	15	12	11	10	13	11
*CAPN10*	6	6	6	6	6	14	2	14	14	12	1	4	5	1	1	10	3	12	8	8
*CCNG1*	14	15	14	14	14	13	15	13	13	15	16	16	16	16	16	13	12	9	14	12
*EEF1A1*	3	4	4	3	3	8	12	7	8	11	13	14	14	11	14	7	7	5	9	6
*EID2*	10	8	10	10	10	7	9	8	7	9	8	13	9	6	10	4	6	8	2	4
*GAPDH*	7	10	9	7	8	5	11	6	5	5	4	6	7	4	6	5	5	4	6	5
*HPRT1*	16	11	16	16	15	12	14	12	12	14	7	12	10	5	9	9	9	7	10	10
*MYC*	15	16	15	15	16	15	1	15	15	8	5	1	3	9	3	16	15	16	16	16
*NAT1*	5	3	5	5	5	11	4	11	11	10	10	11	12	10	12	8	8	11	5	9
*PHB*	12	14	12	12	12	4	5	4	4	4	9	9	11	7	11	15	16	15	15	15
*RABEP2*	13	13	13	13	13	1	6	3	2	2	6	8	8	8	8	14	14	14	12	14
*RPLP0*	1	2	1	1	1	6	10	5	6	6	11	3	1	13	5	3	4	1	4	3
*TBP*	8	9	8	8	9	3	7	1	3	3	3	5	6	3	4	1	2	3	1	1
*TUBB3*	2	1	1	2	2	9	13	9	9	13	14	10	13	14	13	6	10	6	7	7
*UBC*	4	5	3	4	4	2	8	1	1	1	12	7	1	12	7	2	1	1	3	2
*ZNF324B*	9	7	7	9	7	10	3	10	10	7	2	2	4	2	2	11	13	13	11	13

ΔCt ΔCt method, BK - BestKeeper, gN - geNorm, NF - NormFinder, CR - comprehensive ranking

Different reference genes were validated for each developmental stage (*RPLP0* for NSC, *UBC* for eNP, *CAPN10* for NP). Moreover, the most stable expressed gene for the NSC stage (*RPLP0* gene) belongs to the most unstably expressed (13 in the ranking) at the NP stage. None of the genes, *RPL0* (for the NSC), *UBC* (for the eNP) or *CAPN10* (for the NP), have the most stable expression level if we take into account the calculations from all three stages of neural development. In this case, the *TBP* gene is the most stable. In turn, this gene is only eighth in the ranking for the NSC stage (Table 2). Considering all of this data, we suggest using separate reference gene(s) for each developmental stage. Using one for all three stages can lead to false results and misinterpretations.

A similar topic was addressed by the recently published article of Artyukhov and colleagues (2017) [23]. This study cannot be compared directly to our results for several reasons. Artyukhov and colleges (2017) [23] analysed candidates for reference genes for different stages of hiPSC neural differentiation as compared to the analysis performed by our group: they used hiPSC, NSC and neurons (terminally differentiated), while our group analysed populations typical for early neural development: NSC, eNP and NP. The other reason is that only four genes (*ACTB*, *GAPDH*, *HPRT1*, *UBC*) recur in both panels of candidates for reference genes. In addition to different stages of neural development, in both experiments, stem cells were derived from different sources and were cultured under diverse conditions (Table 3).

**Table 3:** Reference gene expression stability values in hiPSC-derived neural stem cells and neural progenitors

	NSC					eNP					NP					NSC + eNP + NP				
Reference gene	ΔCt	BK	gN	NF	CR	ΔCt	BK	gN	NF	CR	ΔCt	BK	gN	NF	CR	ΔCt	BK	gN	NF	CR
*ACTB*	0.58	0.475	0.405	0.426	11.24	2.94	3.352	1.816	2.832	16.00	0.92	0.926	0.742	0.767	15.00	2.50	1.807	1.192	2.223	11.45
*CAPN10*	0.49	0.195	0.282	0.305	6.00	2.19	0.500	1.557	1.996	8.61	0.65	0.401	0.487	0.312	2.11	2.03	0.785	1.448	1.201	7.33
*CCNG1*	0.68	0.500	0.474	0.573	14.24	2.09	2.852	1.465	1.788	13.47	1.01	1.000	0.775	0.879	16.00	2.51	1.856	1.097	2.289	11.84
*EEF1A1*	0.43	0.105	0.211	0.178	3.46	1.66	2.099	0.955	0.983	8.56	0.79	0.741	0.709	0.565	12.94	1.90	1.230	0.780	1.359	6.85
*EID2*	0.54	0.210	0.378	0.395	9.46	1.64	1.722	1.037	0.967	7.71	0.76	0.667	0.594	0.519	8.66	1.73	1.091	0.961	0.669	4.43
*GAPDH*	0.51	0.278	0.356	0.315	8.15	1.49	2.037	0.857	0.657	6.37	0.71	0.444	0.539	0.474	5.09	1.74	1.019	0.680	1.047	4.95
*HPRT1*	0.77	0.444	0.544	0.684	14.57	2.04	2.741	1.387	1.692	12.47	0.75	0.667	0.624	0.511	8.05	1.96	1.312	0.900	1.405	8.68
*MYC*	0.75	0.593	0.511	0.650	15.24	2.46	0.494	1.656	2.297	7.62	0.75	0.105	0.412	0.549	3.41	3.24	2.926	2.163	3.054	15.74
*NAT1*	0.46	0.105	0.240	0.252	4.40	1.94	1.173	1.278	1.599	8.54	0.78	0.654	0.666	0.553	10.72	1.95	1.278	1.345	1.024	7.70
*PHB*	0.58	0.494	0.426	0.431	12.47	1.44	1.333	0.655	0.627	4.23	0.77	0.574	0.649	0.521	8.89	3.17	3.319	2.009	2.953	15.24
*RABEP2*	0.63	0.475	0.446	0.523	13.00	1.36	1.407	0.598	0.337	2.45	0.75	0.494	0.558	0.530	7.44	2.62	2.774	1.810	2.213	13.47
*RPL0*	0.40	0.000	0.000	0.116	1.19	1.51	1.570	0.797	0.705	6.51	0.79	0.401	0.323	0.601	4.55	1.73	0.794	0.584	0.968	2.63
*TBP*	0.51	0.278	0.333	0.336	8.24	1.41	1.519	0.583	0.429	2.82	0.70	0.404	0.525	0.456	4.05	1.66	0.753	0.638	0.639	1.57
*TUBB3*	0.40	0.000	0.000	0.116	1.41	1.73	2.593	1.108	1.153	9.87	0.82	0.645	0.690	0.601	12.63	1.80	1.321	0.838	1.084	7.09
*UBC*	0.45	0.105	0.157	0.235	3.94	1.37	1.685	0.583	0.302	2.00	0.79	0.475	0.323	0.596	5.63	1.70	0.744	0.584	0.848	1.57
*ZNF324B*	0.52	0.195	0.311	0.356	7.94	1.78	0.833	1.198	1.379	7.40	0.67	0.370	0.468	0.385	2.38	2.39	2.140	1.639	1.890	11.96

ΔCt ΔCt method, BK - BestKeeper, gN - geNorm, NF - NormFinder, CR - comprehensive ranking

Artyukhov and colleagues (2017) searched for an optimal set of reference genes for accurate normalisation of qRT-PCR data obtained from studies involving iPSCs, NSCs and mature neurons derived from amniotic fluid samples. They studied 16 genes which are potentially expressed at stable level: *ACTB*, *B2M*, *C1orf43*, *EMC7*, *GAPDH*, *GPI*, *HMBS*, *HPRT1*, *PSMB4*, *REEP5*, *RPL13A*, *SDHA*, *SNRPD3*, *UBC*, *VCP* and *YWHAZ*. The authors applied geNorm and NormFinder algorithms to pick up five reference genes with proved stability in three experiments. They found that accurate normalisation of relative gene expression according to geNorm’s requires two reference genes for the following: hiPSCs (*RPL13A*, *VCP*), NSCs (*HMBS*, *REEP5*) and for mature neurons (*C1orf43*, *HMBS*), while four genes (*C1orf43*, *HMBS*, *PSMB4*, *GAPDH*) for all tested groups of cells [23]. According to NormFinder’s, they indicated that accurate normalisation requires three reference genes (*C1orf43*, *GAPDH*, *YWHAZ*) for iPSCs, two reference genes for NSCs (*PSMB4*, *REEP5*), three reference genes (*ACTB*, *C1orf43*, *GAPDH*) for mature neurons and four genes (*C1orf43*, *HMBS*, *PSMB4* and *YWHAZ*) for all the samples together.

Nevertheless, the general conclusions of the work of Artyukhov and colleagues (2017) [23] and our results are consistent to show that cell populations at different developmental stages require their own, customised set of reference genes.

In another study, Holmgren and colleagues (2015) [1] analysed candidates of reference genes for human embryonic stem cells (hESC) and human-induced pluripotent stem cells (hiPSCs) differentiated to ecto-, meso- and endoderm; thus, they used different developmental stages of hiPSCs as compared to our study. The most stable genes indicated by Holmgren et al. (2015) (*EID2*, *ZNF324B*, *CAPN10*, *RABEP2*) were included in our panel of candidate reference genes. Vossaert and colleagues s (2013) [22] analysed candidates for reference genes for hESC after induction of ectodermal differentiation. Some genes used in the study (*TBP*, *GAPDH*, *HPRT1*, *ACTB*, *UBC*) were included to our panel of our reference gene candidates because of the early developmental aspect of the investigation; however, this report also did not include stages of hiPSC neural differentiation that were analysed in our study [24]. Finally, our group has concentrated only on the early steps of neural development (NSC, eNP, NP), which are difficult to distinguish and have not been compared in the reports of other groups discussed above. The articles discussed above and our results provide the useful method for the identification of genes appropriate for test panels in many different experimental set-ups.

In summary, the panel of 16 candidates for reference genes proposed here can be successfully used for the prediction of reference genes for three different hiPSC-derived stages of neural development. We found that the NormFinder software package was the most robust method of evaluating reference gene expression stability, because it takes into consideration both the inter- and intra-variability during stabilisation assessment. Our results also demonstrate that no single reference gene or reference gene combination is suitable for all developmental stages analysed. Therefore each stage of development requires its own panel for optimal normalisation of RT–qPCR data. Furthermore, the results point to the importance of using different algorithms in this type of analysis to guarantee strong confidence in the correct choice of reference genes.

In conclusion, the results of our work emphasise the importance of proper selection of reference genes and the need for the customised validation of their stability in studies in stem cell research.

## Electronic Supplementary Material

Supplementary data_1: Candidates for reference genes Gene Ontology (GO) enrichment analysis (XLS 366 kb)

Supplementary data_2: Determination of the optimal number of control genes for normalization calculated by NormFinder. The lowest expression value of the Acc. S.D. shows the optimal number of genes for normalization (grey box): (A) NSC, (B) eNP, (C) NP, (D) NSC+eNP+NP. (PNG 206 kb)

High resolution image (TIF 105630 kb)

Supplementary data_3: Reference gene ranking order in hiPSC-derived neural stem cells and neural progenitors in the form of a heat map. Columns in the heat map represent tested genes, and the rows represent methods for gene validation. Colour intensity values correspond to order in the ranking. (XLSX 12 kb)

Supplementary data_4: Reference gene expression stability values in hiPSC-derived neural stem cells and neural progenitors in the form of a heat map. Columns in the heat map represent tested genes, and the rows represent cell line. Colour intensity values correspond to expression stability value for each method. (XLSX 14 kb)

## Abbreviations

*ACTB*: Actin beta
*B2M*: Beta-2-microglobulin
*CAPN10*: Calpain 10, calcium-activated neutral proteinase 10
*CCNG1*: Cyclin G1
*C1orf43*: Chromosome 1 open reading frame 43
*EEF1A1*: Eukaryotic translation elongation factor 1 alpha 1
*EID2*: EP300 interacting inhibitor of differentiation 2
*EMC7*: *ER* membrane protein complex subunit 7
eNP: early Neural Progenitors
*GAPDH*: Glyceraldehyde-3-phosphate dehydrogenase
*GPI*: Glucose-6-phosphate isomerase
hESC: human Embryonic Stem Cells
hiPSC: human induced Pluripotent Stem Cells
HKGs: Housekeeping genes, reference genes, endogenous control
*HPRT1*: Hypoxanthine phosphoribosyltransferase 1
*HMBS*: Hydroxymethylbilane synthase
*LIN28*: LIN-28 Homolog A
*MAP2*: Microtubule-associated protein 2
*MYC*: V-myc avian myelocytomatosis viral oncogene homolog
*NANOG*: Homeobox protein NANOG
*NAT1*: N-acetyltransferase1
*NES*: Nestin
NP: Neural Progenitors
NSC: Neural Stem Cells generated from hiPSC
*PAX6*: Paired box 6
*PHB*: Prohibitin
*PSMB4*: Proteasome subunit beta 4
*POU5F1*: POU class 5 homeobox 1, OCT4, OCT3/4
*RABEP2*: Rabaptin, RAB GTPase binding effector protein 2
*REEP5*: Receptor accessory protein 5
*RPLP0*: Ribosomal protein lateral stalk subunit P0
*RPL13A*: Ribosomal protein L13a
RT-qPCR: quantitative real-time Polymerase Chain Reaction
*SDHA*: Succinate dehydrogenase complex subunit A
*SNRPD3*: Small nuclear ribonucleoprotein D3 polypeptide
*SOX1*: SRY-box 1
*SOX2*: Transcription factor SOX2
*SV40LT*: Simian virus 40 large T antigen
*TBP*: TATA-box binding protein
*TUBB3*: Tubulin Beta 3 Class III
*UBC*: Ubiquitin C
*VCP*: Valosin containing protein
*YWHAZ*: Tyrosine 3-monooxygenase/tryptophan 5-monooxygenase activation protein zeta
*ZNF324B*: Zinc finger protein 324B
