# Supplementary figures and images for: Reference Gene Validation via RT–qPCR for Human iPSC-Derived Neural Stem Cells and Neural Progenitors
Source: Mol Neurobiol. 2019 Mar 29;56(10):6820–32. doi: 10.1007/s12035-019-1538-x (PMC6728297; doi:10.1007/s12035-019-1538-x)

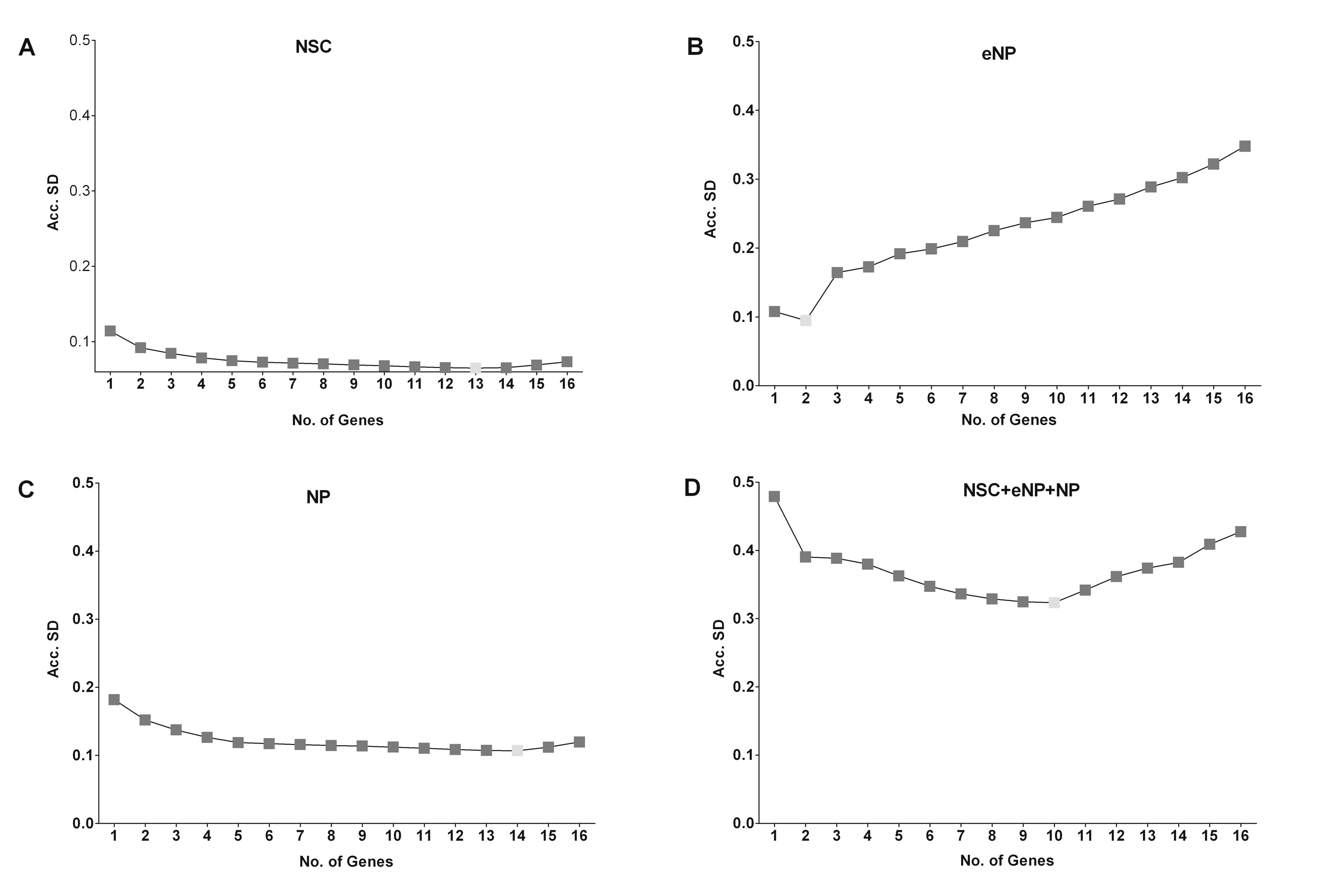

Supplement: Supplementary file 2 — Determination of the optimal number of control genes for normalization calculated by NormFinder. The lowest expression value of the Acc. S.D. shows the optimal number of genes for normalization (grey box): (A) NSC, (B) eNP, (C) NP, (D) NSC+eNP+NP. (PNG 206 kb) [file 12035_2019_1538_Fig7_ESM.png]
